# Supplementary material for: Attentional priorities drive effects of time pressure on altruistic choice
Source: Nat Commun. 2020 Jul 15;11:3534. doi: 10.1038/s41467-020-17326-x (PMC7363879; doi:10.1038/s41467-020-17326-x)
Supplement: Supplementary file 1 — Supplementary Information [file 41467_2020_17326_MOESM1_ESM.pdf]

Supplementary Information for
"Attentional priorities drive effects of time pressure on altruistic choice"

Yi Yang Teoh, Ziqing Yao, William A. Cunningham, Cendri A. Hutcherson

Supplementary Notes

Supplementary Note 1

To confirm the robustness of the behavioral findings reported in Study 1, we ran three behavioural replications of Study 1. Replication studies 1A (N=34) and 1B (N=31) resembled our Study 1, with the following differences. Participants completed 200 trials of the modified dictator game as in the Study 1. However, to ensure that effects were not driven by the left-right positioning of decision attributes ($Self & $Other), attributes were presented vertically arranged in the center of the screen with either $Self or $Other on top. In Study 1A (1B), the high time pressure response window was 2s (1.5s). In both studies, participants in the free response condition were only instructed to choose within 10s and not explicitly encouraged to delay responses. Finally, in Study 1A, the scale of the monetary offers range from $0 to $20. Generous choices did not vary as a function of the study variant coded as a dummy variable (b = -0.0463, SE = 0.2588, z = -0.179, p = .86) or moderate the effect of the time pressure conditions (b = 0.0407, SE = 0.0768, z = 0.530, p = .60). We thus collapsed results of these two studies for analyses. In replication Study 2, participants (N=49) completed 200 trials of the modified dictator game, with $Self and $Other displayed on the right and left of the screen exactly as in the Study 1, but in the absence of eye-tracking to ensure that effects were not driven by a measurement of eye-movements. All other details of the experimental paradigms were identical to Study 1.

As expected, we replicated the effects of time pressure on reaction times (Replication Study 1: M_{high}=0.799s, M_{low}=1.071s, SE_{diff}=0.033s, t_{64}=-8.284, p<.001; Replication Study 2: M_{high}=0.742s, M_{low} =3.420s, SE_{diff}=0.117s, t_{48}=-22.807, p < .001). However, while Study 1 reported a small but significant decrease in generosity under time pressure, this effect failed to

replicate in our replication studies (Replication Study 1: $M_{high} = 0.440$, $M_{low} = 0.437$, $SE_{diff} = 0.007$, $t_{64} = 0.4114$, $p = .68$; Replication Study 2: $M_{high} = 0.424$, $M_{low} = 0.442$, $SE_{diff} = 0.011$, $t_{48} = -1.475$, $p = .15$).

However, importantly, we replicated the main correlational analyses demonstrating robust individual differences in changes in generosity under time pressure. We show again that generosity under low time pressure predicted changes in generosity with time (Replication Study 1: Pearson's $r = -0.286$, $t_{63} = -2.370$, $p < .05$; Replication Study 2: Pearson's $r = -0.300$, $t_{47} = -2.154$, $p < .05$) and generosity under low time pressure was not associated with changes in generosity (Replication Study 1: Pearson's $r = 0.004$. $t_{63} = 0.0322$, $p = .97$; Replication Study 2: Pearson's $r = 0.083$, $t_{47} = 0.570$, $p = .57$). These consistent effects strongly support our interpretation that biases in early attention may drive choice biases under time pressure that become attenuated with more time.

Supplementary Note 2

In this paper, we proposed that time pressure produced choice biases that differed between individuals by showing that extreme choice biases under time pressure were attenuated with time. We demonstrated this effect by correlating the proportion generosity under high time pressure with change in proportion generosity from high time pressure to low time pressure (Study 1: Pearson's r = -0.313, t_{58} = 2.513, p = .0148; Replication Study 1: Pearson's r = -0.286, t_{63} = -2.3702, p = .021; Replication Study 2: Pearson's r = -0.300, t_{47} = -2.1537, p = .036).

To rule out the possibility that these effects were due to regression towards the mean, we conducted three additional analyses. First, to illustrate that our measures of generosity were relatively precise, we conducted tests of reliability across blocks of our experiments since regression to the mean is most likely to occur when variability in the measures is high. Notably, we found excellent reliability across blocks in generosity estimates for each condition in Study 1 (High time pressure: Cronbach's α [95% CI] = 0.94 [0.91, 0.96]; Low time pressure: α = 0.93 [0.90, 0.96]), Replication Study 1 (High time pressure: α = 0.93 [0.91, 0.96]; Low time pressure: α = 0.92 [0.90, 0.95]) and 2 (High time pressure: α = 0.95 [0.93, 0.97]; Low time pressure: α = 0.95 [0.92, 0.97]). This strongly suggests that measures of generosity within the paradigm are stable and vary minimally across measurement within an individual, reducing the likelihood of regression towards the mean.

Secondly, we re-ran the same correlational analyses we performed on the full set of trials investigating the association between generosity under high time pressure and change in generosity with time, but now separately for each block. Given that regression to the mean emerges as a function of random variability in measurement, it would be unlikely to produce consistent effects. Instead, we found overwhelming consistency in the pattern of effects across

all blocks (Study 1: mean Pearson's r = -0.366, all *ps* < .01; Replication Study 1: mean Pearson's r = -0.401, all *ps* < .05; Replication Study 2: mean Pearson's r = -0.358, all *ps* < .05). Given the robustness of this correlation, we think it is highly unlikely that random variability in estimates resulting in regression to the mean would exhibit such consistency across multiple tests and experiments.

Finally, we also performed an even stronger test of regression to the mean vs. our favoured hypothesis. Assumptions of regression to the mean suggest that the strongest drivers of the effect would be the most extreme predictors (since these are the observations that can shift the most towards the mean). In contrast, our prioritized attention model suggests that the most extreme individuals (ones who attend 100% of the time to self or to other exclusively) should actually be *least* likely to shift in a more moderate direction, since they are better able to implement their true preferences. It should actually be the individuals who give at least *some* weight to the secondary attribute, and attend to it when given enough time, who show the most change. Thus, the regression to the mean explanation suggests that observed associations between generosity and change due to time pressure should get weaker when the most extreme individuals are excluded, whereas our model predicts that this association should stay the same, or if anything get stronger. To test these two distinct possibilities, we ran a follow-up analysis excluding participants who were < 25% generous or > 75% generous under time pressure. Instead of attenuating the original effect (Study 1: Pearson's r = -0.313, t_{58} = 2.513, p = .0148; Replication Study 1: Pearson's r = -0.286, t_{63} = -2.3702, p = .021; Replication Study 2: Pearson's r = -0.300, t_{47} = -2.1537, p = .036), we find that the effect becomes stronger when excluding the participants most likely to regress towards the mean (Study 1: Pearson's r = -0.490, t_{35} = -3.326, p = .0021; Replication Study 1: Pearson's r = -0.408, t_{52} = -3.221, p = .0022; Replication Study

2: Pearson's r = -0.522, t_{32} = -3.459, p = .0016).

This effect is fully consistent with our subsequent analyses that show individuals' gaze-biases to be particularly important in mediating this effect. For example, extremely selfish individuals typically make consistently selfish choices under low time pressure as an expression of their selfish social preferences. Under high time pressure, they are also likely to search exclusively for their own outcomes first, ensuring that their selfish preferences are sustained and expressed. Thus, we observe no change in their behavior. In contrast, moderately selfish individuals, while still preferring to maximize their own outcomes, also give some attention to others' outcomes under low time pressure. Under high time pressure however, because they are more likely to search for their own outcomes first, and then have little time to acquire and process the other person's outcomes, their choices become more extreme reflections of their underlying preferences.

Furthermore, our model cross-validation reported in the main paper strongly suggests that individual-level parameters estimated from half of the data is sufficient to predict patterns of change in choice behavior under time pressure in an out-of-sample dataset. These systematic associations between social preferences, attention and choice would be highly unlikely in an account of choice that simply assumes regression to the mean.

Supplementary Note 3

In this paper, we conducted continuous analyses of eye-gaze to illustrate the effects of time pressure on early attention and its subsequent influences on generous choice. To demonstrate the robustness of these results, we attempted to replicate all relevant analyses using first fixation position as a measure of early attention. Using a fixation threshold of 100ms, we extracted which information – self or other – participants looked at first in each trial. Logistic mixed-effects regression predicting first fixation position (0: other-information, 1: self-information) by time pressure condition reveals a significant main effect of time pressure (b = 0.783, SE = 0.062, z = 12.702, p < .001, see Supplementary Fig. 2). Specifically, we found that participants were biased towards self-information under high time pressure (b_0 = 1.395, SE = 0.332, z = 4.198, p < .001) but not when they had more time to decide (b_0 = 0.612, SE = 0.331, z = 1.848, p = .065), replicating our findings reported with continuous gaze measures.

Additionally, we found marginally a significant correlation between fixation biases (proportion of first fixations on self-information – proportion of first fixations on other-information) under high time pressure and changes in these biases from high to low time pressure (Pearson's r = -0. 225, t_{55} = -1.710, p = .093). Here, we similarly found that individual differences in first fixation biases emerged under time pressure and were mitigated with time, consistent with analyses using continuous gaze measures. Unlike analyses using continuous gaze, we also found a significant correlation between fixation biases under low time pressure and changes in these biases from low to high time pressure (Pearson's r = -0.313, t_{55} = -2.445, p = .018). We speculate that this association is due to individuals who already possess strong biases under low time pressure becoming more noisy in their attention-allocation and regressing to the mean. However, this is not inconsistent with our hypotheses regarding the emergence of unique

attentional biases under time pressure. Moreover, it contradicts previous work suggesting individuals become more extreme in their biases under time pressure.

Most importantly, we replicate the effects of attention on generous choice. A logistic mixed-effects model predicting generosity revealed a marginally significant interaction between time pressure and fixation biases (*b* = -0.133, SE = 0.0709, z = -1.878, p = .060, see Supplementary Fig. 3). Specifically, individual differences in fixation biases strongly predicted generosity under high time pressure (*b* = -0.345, SE = 0.115, z = - 2.989, p = .003), but not as strongly under low time pressure (*b* = -0.212, SE = 0.113, z = - 1.872, p = .061).

Thus, we found robust support for our model suggesting that attention drives time pressure's effect on generosity whether we used continuous temporal measures of early attention or fixation-based measures.

Supplementary Note 4

We argue in this paper that attentional deployment may explain changes in choice resulting from time pressure. However, alternative models have also been proposed. In particular, Chen & Krajbich (2018) recently proposed a model of biased decision-making in which individuals begin with a predisposition to respond generously or selfishly, which the authors capture using a "generosity bias" parameter for the starting point of the evidence-accumulation process[1]. In other words, some people may begin with a bias to respond selfishly, counteracting such bias only if sufficient evidence accumulates in favour of a generous response. Generous individuals start with the reverse bias. Using a static model of evidence-accumulation, Chen & Krajbich found that fitted values of a generosity bias in free response conditions were correlated with whether an individual became more extreme under time pressure or more moderate under time delay.

Although this model provides a simple and attractive mechanism for implementing a form of dual-process model, we suspected that attentional gaze dynamics could, if left unmodeled, be recovered as choice biases. Here, we provide two pieces of evidence that this may be the case.

First, we confirmed through simulations that fitting a static model with a starting bias to a true model in which early gaze-biases the drift would, indeed, result in erroneous attribution of changes in time pressure to a starting bias. To do so, we simulated 200 choice trials (100 high, 100 low time pressure) for each of 20 hypothetically selfish ($w_{self} = .03$, $w_{other} = .02$) and 20 hypothetically generous ($w_{self} = .02$, $w_{other} = .03$) participants, who attended either to self or other information first and oscillated between the information sources every 200ms. In these simulations, subjects' social preference and attentional priorities were orthogonal. Thresholds for

all simulations were fixed at $b = .16$, and time pressure was simulated using a collapse rate $d = 0$ in the low time pressure condition and $d = .8$ in the high time pressure condition. We added 300ms to the resulting reaction times to account for motor and perceptual processing. Importantly, none of the simulations included true starting point biases. In other words, we simulated a model in which eye-gaze was the true driver of changes in choice under time pressure, and examined how such a mechanism influenced recovered parameters from an incorrect, static model of choice.

To this end, we used the Hierarchical Drift-Diffusion Modelling **(**HDDM**)**[2] package to implement a *social bias model* including weights on self, other, *ndt, b, stbias* and *genbias* paramaters. The *social bias model* assumes that changes in generosity under time pressure reflect limitations on static evidence-accumulation, (i.e. reductions in *b*) that exacerbate the effects of existing social predispositions (i.e. *genbias*). Thus, we fixed the weights and biases across time pressure conditions during model-fitting.

Results suggest that the fitted model explained the simulated data well, accurately predicting inter-individual differences in changes in generosity and RTs across time pressure conditions (Generosity: Pearson's $r = 0.702$, $t_{38} = 6.087$, $p < .001$; RTs: Pearson's $r = 0.487$, $t_{38} = 3.44$, $p < .01$), and intra individual differences in choices across different trial types under both high and low time pressure (High time pressure: mean Pearson's $r = 0.852$, SE = 0.0145, $t_{39} = 58.70$, $p < .001$; Low time pressure: Pearson's $r = 0.913$, SE = 0.0070, $t_{39} = 130.00$, $p < .001$). The model also predicted intra-individual differences in RTs moderately well under both high and low time pressure (High time pressure: mean Pearson's $r = 0.477$, SE = 0.0482, $t_{39} = 9.91$, $p < .001$; Low time pressure: Pearson's $r = 0.755$, SE = 0.216, $t_{39} = 35.00$, $p < .001$,

Supplementary Fig. 4). Thus, our simulations suggest that a researcher fitting a static model would conclude that the model fit the data reasonably accurately.

Turning to the recovered parameters, we found that the static social bias model accurately recovered the true simulated weight parameters, w_{self} (Pearson's $r = 0.882$, $t_{38} = 11.596$, $p < .001$) and w_{other} (Pearson's $r = 0.911$, $t_{38} = 13.679$, $p < .001$). However, a logistic mixed-effects model erroneously suggested that the retrieved *genbias* parameters interacted with time pressure to predict generosity (Interaction effect: $b = 6.959$, SE = 1.357, $z = -5.130$, $p < .001$), with *genbias* parameters more predictive of generosity under high time pressure (simple effect of *genbias*: High Time Pressure: $b = 6.313$, SE = 2.419, $z = 2.610$, $p < .01$; Low Time Pressure: $b = -0.646$, SE = 2.392, $z = -0.270$, $p = .79$). While this replicated findings reported by Chen & Krajbich, we found that the average *genbias* parameters were also highly correlated with the initial piece of information attended (Pearson's $r = 0.875$, $t_{38} = 11.171$, $p < .001$), suggesting that generosity biases were largely tracking variance induced by attentional dynamics rather than true, pre-determined response biases.

The above simulations suggest that, if the true model of choice behaviour involves dynamic changes in biased evidence-accumulation due to eye-gaze, a static model might mis-identify eye-gaze with starting point biases. We thus sought to determine whether such a correlation existed in our own data. To test this possibility, we also fit the *social bias model* to choice and reaction time data within our experiment using the HDDM package. All fits of the data using HDDM excluded trials in which no responses were recorded.

As expected, generosity biases recovered by the model were correlated with participant's average early gaze-biases (Pearson's $r = -0.460$, $t_{48} = -3.596$, $p < .001$), Furthermore, as in the simulated data, a logistic mixed-effects regression also identified *genbias* parameters interacting

with time pressure to predict generosity in the experimental data (*b* = -3.149, SE = 1.384, *z* = -2.276, p < .05), erroneously suggesting that generosity under time pressure was more strongly driven by intuitive social biases (simple effect of *genbias*: High Time Pressure: *b* = 6.972, SE = 1.622, *z* = 4.298, p < .001; Low Time Pressure: *b* = 3.824, SE = 1.577, *z* = 2.424, p < .05). Taken together, our results provide support for the idea that early gaze-biases, if not explicitly modeled, could yield erroneous identification of a social response bias.

Supplementary Note 5

To investigate the relationship between one-shot continuous generation games[3,4], that have shown intuitive biases to drive behavior under time pressure, and iterative binary choice games that we used in Study 1, we used both tasks to measure generous behavior in Study 2 (see Supplementary Fig. 1 & 3). Participants in Study 2 were randomly assigned to one of two groups and completed 5 trials of an anonymous one-shot dictator game prior to the binary-choice tasks. In this section, they were given varying sums of money ($100, $50, $20, $10, $2) and asked to indicate how much they would allocate to their partner on a sliding scale (see Supplementary Fig. 5). In one group, participants had to make these choices within 10s (high time pressure: N=100) while participants in the other group had to consider the choice for 10s before they were allowed to submit their choice (low time pressure: N=100). Participants always encountered the trial where the sum was $100 first. The subsequent trials were randomly ordered. We only report the results of analyses conducted on only the first trial of the one-shot game, but analyses of the average generosity across the five trials show similar patterns of effects

Importantly we find that generosity measured in the binary choice dictator game was associated with generosity measured in the one-shot analog (Pearson's $r = 0.447$, $t_{198} = 7.029$, $p < .001$). Additionally, to ensure that time pressure manipulations that vary across these two measures (continuous generation: < 10s; binary choice: < 1.5-2s) exert their effects on the same underlying process, we looked for associations between generosity in the binary choice games and continuous games for matching conditions. Specifically, generosity in the binary-choice game under *high time pressure* was highly predictive of generosity in the continuous one-shot game under *high time pressure* (Pearson's $r = 0.493$, $t_{98} = 5.614$, $p < .001$). Similarly, generosity in the binary-choice game under *low time pressure* was highly predictive of generosity in the

continuous one-shot game under *low time pressure* (Pearson's $r = 0.413$, $t_{98} = 4.491$, $p < .001$). However, although correlations were technically higher in the matching conditions, they were not significantly higher than those in the non-matching conditions (binary *low* predicting continuous *high*: Pearson's $r = 0.456$, $t_{98} = 5.072$, $p < .001$; Difference in dependent correlations: Fisher's $t = 0.95$, $p = .34$; binary *high* predicting continuous *low*: Pearson's $r = 0.398$, $t_{98} = 4.293$ $p < .001$; Difference in dependent correlations: Fisher's $t = -0.34$, $p = .73$), likely due to high within-individual correlations between generosity in the binary-choice game for both low and high time pressure conditions (Pearson's $r = 0.892$, $t_{198} = 27.768$, $p < .001$). Together, these results suggest that the different paradigms tap into a similar construct of social preferences and process of choice.

Supplementary Note 6

To investigate whether the low time pressure condition prompted individuals to more extensively deliberate about choice attributes prior to choosing, we conducted additional analyses on the eye tracking data. Firstly, we find that participants made a larger overall number of fixations to self and other information (Poisson mixed-effects regression on number of fixations per trial: $b_{\text{low- high time pressure}} = 1.062$, SE.= 0.0431, $z = 24.60$, $p < .001$). Additionally, the durations of those fixations were longer (linear mixed-effects regression on log-transformed average duration of fixations per trial: $b_{\text{low- high time pressure}} = 0.328$, SE.= .0259, $t_{55} = 12.7$, $p < .001$), when they had more time. This suggests that participants processed more choice information and/or processed the information more fully. Moreover, if people simply stopped deliberating at some point in the free time condition, we would expect a marked rise in fixations in non-AOI regions of the screen over time, and yet this is not what we observe. Gaze remains predominantly fixated on either self or other information until ~7s where more than 95% of trials have terminated, suggesting extended deliberation of both self and other outcomes under time-delay (Supplementary Fig. 6).

Supplementary Note 7

To rule out the effects of block order in explaining our pattern of effects, we re-ran all major analyses for a subset of data, blocks 4 through 7. Since all participants encountered low then high time pressure blocks in an alternating fashion, this subset of data reverses the block order. Importantly, we replicated both our finding that time pressure leads to slightly more selfish behavior (M = 0.0547, t_{59} = 3.32, p = 0.0016, see Supplementary Fig. 7 for block averages) and that these effects vary as a function of individual differences where generosity under time pressure predicts changes in generosity with time (Pearson's r = -0.326 t_{58} = -2.624, p = 0.011). Specifically, generous individuals under time pressure became less generous with time while selfish individuals under time pressure became less selfish. We also find evidence for generosity under low time pressure predicting change in generosity (Pearson's r = - 0.269, t_{58} = -2.13, p = 0.038). However, these effects were primarily driven by 2 outlier points (> 2SD above/below mean). The correlation excluding these points failed to reach significance (Pearson's r = -0.200, t = -1.53, df = 56, p = 0.132).

For measures of gaze-biases, we similarly replicated both effects of time pressure on attention and the effect of individual differences. We found that time pressure increased self-oriented gaze-biases under time pressure (M = 0.159, t_{56} = 4.556, p < .001, see Supplementary Fig. 8 for block by block means). More importantly, individual differences in biases under time pressure become attenuated with time (Pearson's r = -0.600, t_{55} = -5.567 , p < .001) while we find no evidence of biases under low time pressure predicting changes in these biases (Pearson's r = 0.122, t_{55} = 0.908, p = 0.37).

Finally, we replicated the main effect of gaze-biases on choice such that looking at self-outcomes first predicted less generous choices (b = -0.498, SE = 0.202, z = -2.470, p = 0.0135,

semi-partial R^2 = 0.082). As expected, we also found a main effect of time pressure on choice (b = -0.075, SE = 0.0367, z = -2.031, p = 0.042, semi-partial R^2 = 0.008) such that individuals became more selfish under time pressure. Notably, we also found a marginally significant interaction between early gaze and time pressure (b = -0.174, SE = 0.0963, z = -1.804, p = 0.071, semi-partial R^2 = 0.014), replicating our findings in the main manuscript, showing that early gaze has a stronger effect on choice under high time pressure compared to low time pressure.

Taken together, these analyses suggest that our findings are robust and are unlikely to be due to block order.

Supplementary Figures

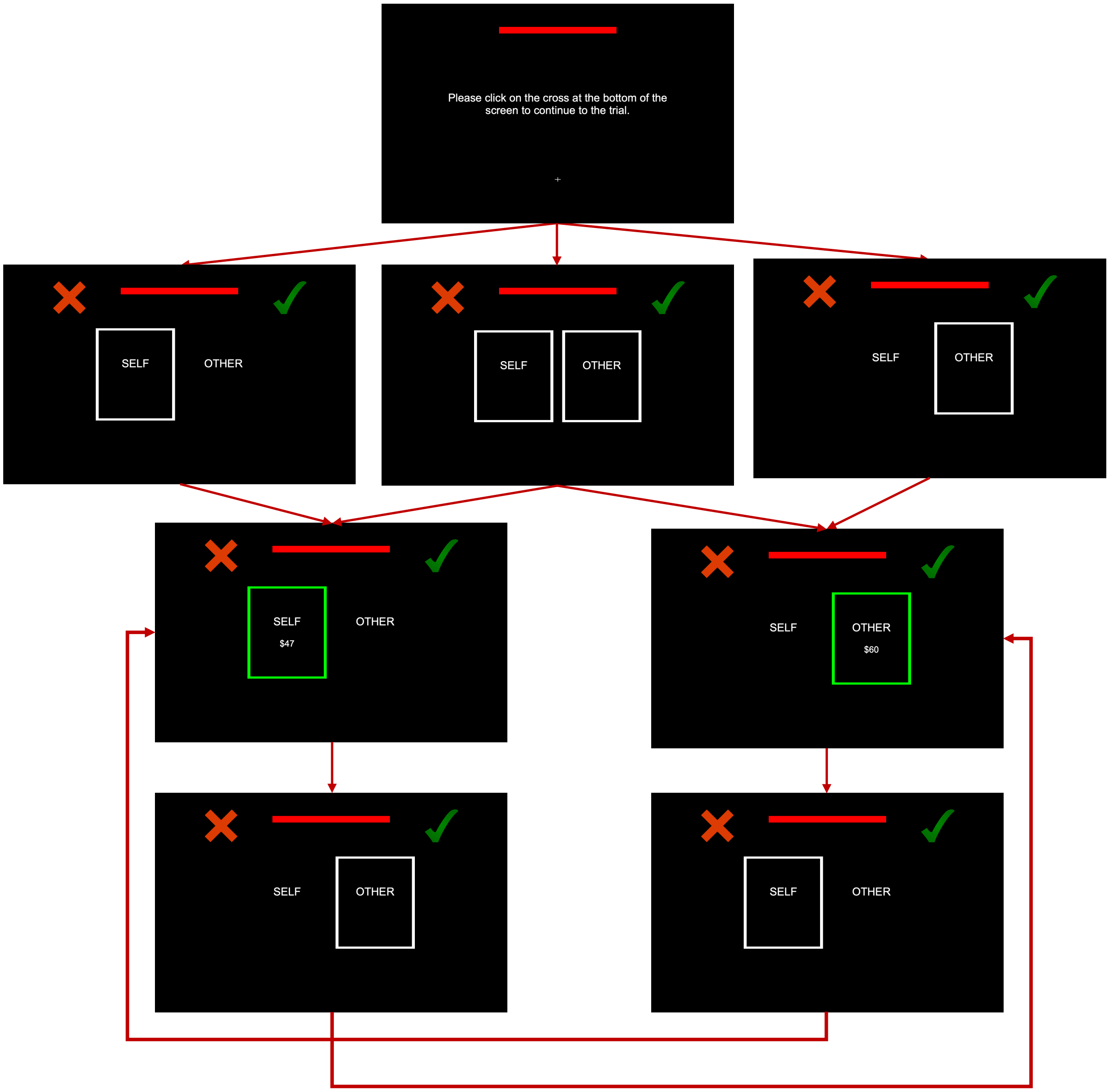

Supplementary Figure 1

Study 2 modified dictator game schema.

Participants were forced to access self-outcomes in one-third of the trials, forced to access other-outcomes in another third of the trials and were allowed to choose which outcomes (self or other) they wished to look at. The available information was indicated by a white border and clicking within the border revealed the respective outcomes for a short period of time. The other piece of information that was previously not accessed was then made available and participants oscillated between the two pieces of information until they made a choice or the time limit had elapsed.

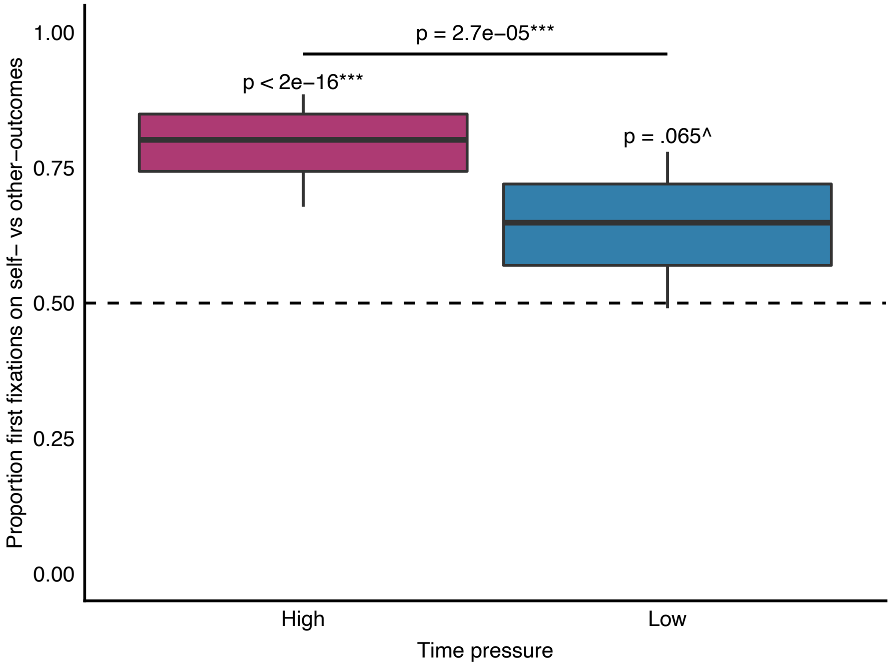

Supplementary Figure 2
Effects of time pressure on mean biases in early attention.
Central line in the boxplot indicate the mean first fixation bias towards self- relative to other-information with the upper and lower boundary of the box reflecting one standard error above and below the mean while the whiskers illustrate the 95% confidence interval of the estimate. The dashed line denotes an unbiased fixation pattern (i.e. equal probability of fixating on self-information or other-information first). N = 57 subjects were included in this paired t-test. ^p < .10,*p < .05, **p < .01, ***p < .001

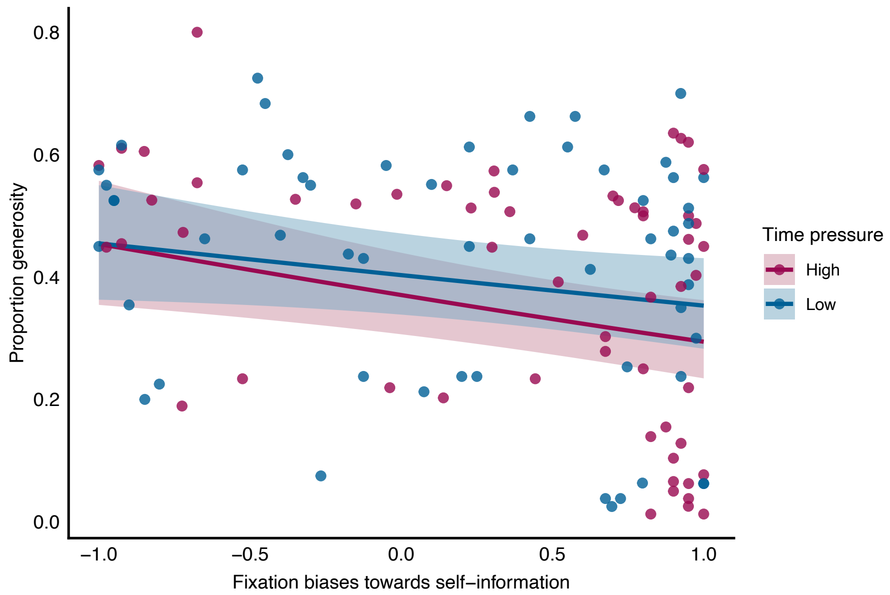

Supplementary Figure 3

Time pressure moderates the effects of fixation biases on generosity.

Each point represents a single subject (N = 57), colored by time pressure condition. Solid lines represent the predicted group averages extracted from the general linear model. Shaded regions represent the 95% confidence interval of the predicted values, colored by time pressure condition.

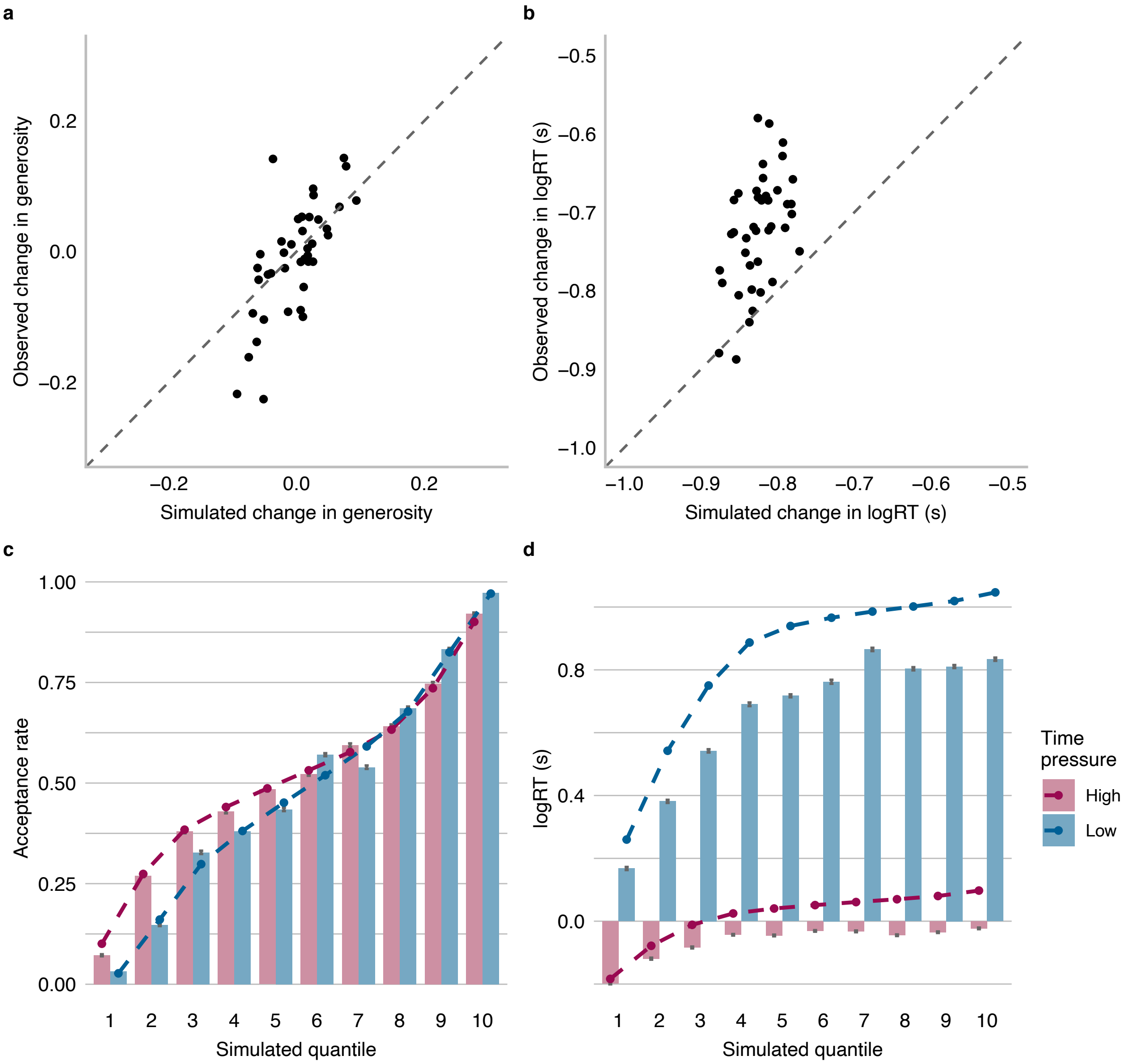

Supplementary Figure 4

Simulation accuracy of the simple DDM.

Simulated versus observed individual differences in **a**) change in proportion generosity under time pressure, and **b**) change in logRT under time pressure. Each point represents a subject (N=40), and points on the dashed line indicate perfect simulation of the model. Bottom panels illustrate simulated versus observed intra-individual differences (N=40 subjects) in **c**) acceptance of proposed offers, and **d**) logRT, binned into ten model-predicted quantiles within individuals. Column height represents the observed group averages with error bars denoting one standard error above and below the group averages. Points on the dashed line represent the simulated averages within these quantiles across individuals.

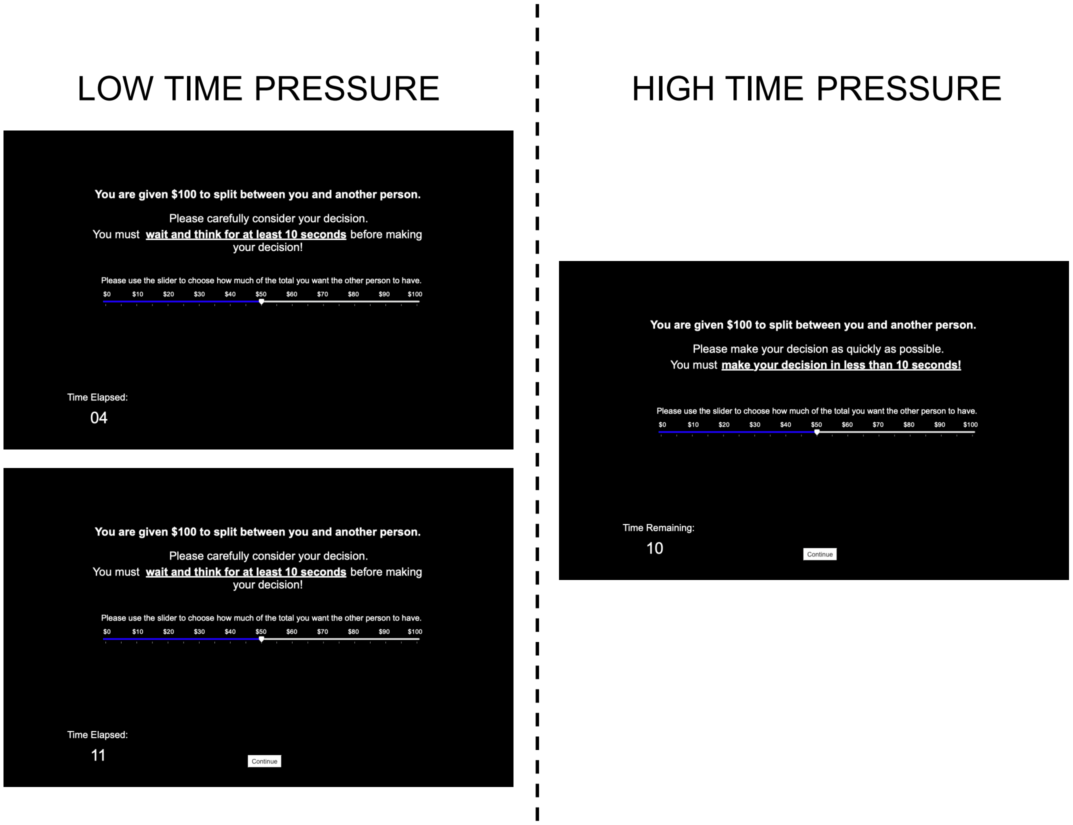

Supplementary Figure 5

Study 2 task schema of a one-shot continuous dictator game under low and high time pressure. The total sum of money given to split on each of the five trials varied ($100, $50, $20, $10, $2). The scale was always initialized to the an even split and the continue button only appeared in the low time pressure condition after 10s have elapsed.

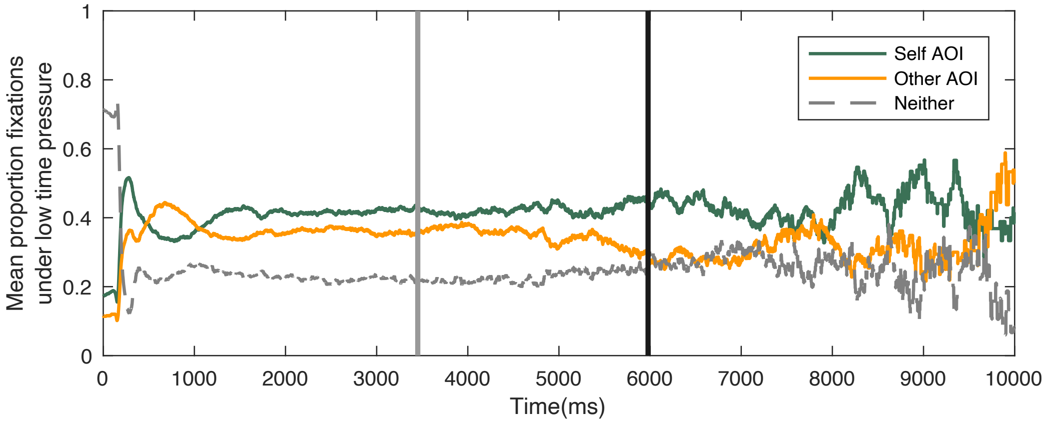

Supplementary Figure 6

Attention dynamics of altruistic choice across time.

Millisecond-to-millisecond proportion of gaze directed to Self AOI, Other AOI or neither under low time pressure, colored by AOI. The vertical lines represent points at which > 50% of trials (grey) and > 95% of trials (black) have terminated.

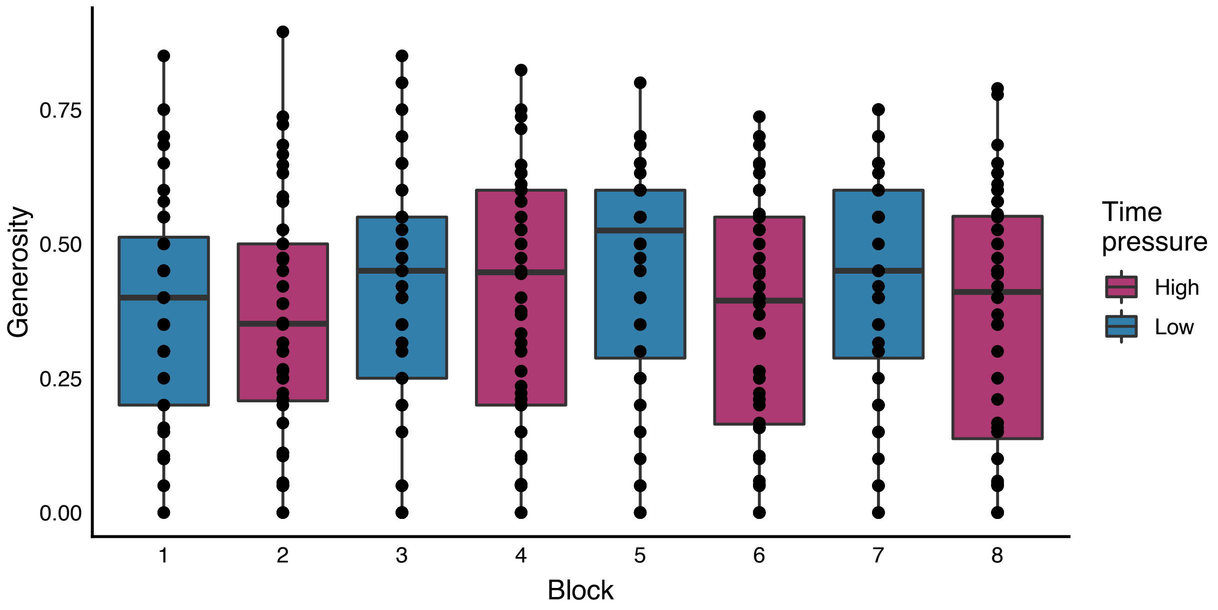

Supplementary Figure 7

Average generosity by block colored by time pressure condition.

Each point represents a single subject (N=60). Central line of the box plot indicates the average generosity while the upper and lower bounds indicate one standard error above and below the mean. The whiskers represent the 95% confidence interval of the mean estimate.

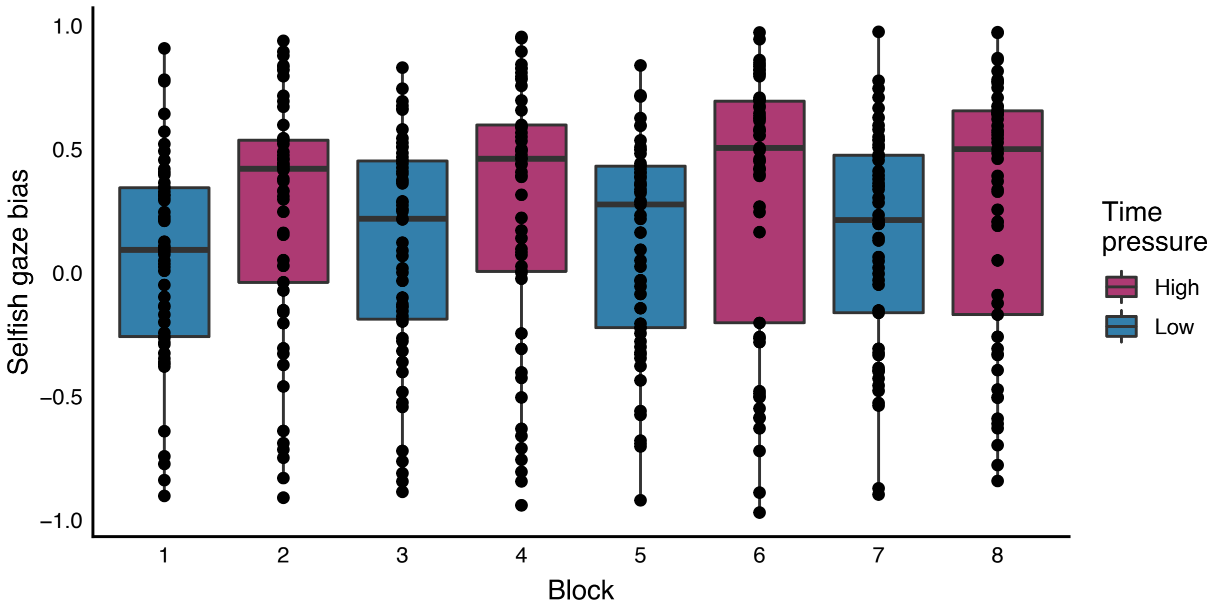

Supplementary Figure 8

Average selfish gaze-bias by block colored by time pressure condition.

Positive values indicate a selfish gaze-bias while negative values indicate an other-oriented gaze bias. Each point represents a single subject (N=57). Central line of the box plot indicates the average generosity while the upper and lower bounds indicate one standard error above and below the mean. The whiskers represent the 95% confidence interval of the mean estimate.

Supplementary Tables

Supplementary Table 1: Logistic mixed-effects model showing individual differences in early gaze predicting generosity.

**Model predicting generosity**					
**Predictors:**	**Statistics**				
	**b**	**SE**	**z**	**two-tailed p**	**semi-partial R^2**
Intercept	-0.4327	0.1367	-3.165	0.00155**	-
Early gaze-biases (+: selfish, -: prosocial)	-0.4944	0.1688	-2.930	0.00339**	0.051
Time pressure (1: High, -1: Low)	-0.0706	0.0268	-2.637	0.00837*	0.004
Late gaze-biases (+: selfish, -: prosocial)	-0.4562	0.2101	-2.171	0.02989*	0.013
Early gaze-biases × Time pressure	-0.1505	0.0716	-2.103	0.03545*	0.005
Late gaze-biases × Time pressure	0.2128	0.1534	1.387	0.16542	0.003

Supplementary Table 2: Pairwise comparison of gaze-informed ADDM parameters between low and high time pressure conditions.

**Parameters**	**M_{high}**	**M_{low}**	**M_{diff}**	**SE**	**t**	**df**	**two-tailed p (uncorrected)**
w_{self}	0.0136 (0.0137)	0.0087 (0.0062)	0.00486	0.00146	3.335	49	0.0016
w_{other}	0.0044 (0.0180)	0.0036 (0.0066)	0.00075	0.00199	0.378	49	0.7068
$w_{fairness}$	0.0066 (0.0076)	0.0031 (0.0027)	0.00348	0.00091	3.849	49	0.0003
b	0.322 (0.104)	0.396 (0.078)	-0.0734	0.0157	-4.661	49	< .0001
d	1.464 (0.420)	0.203 (0.098)	1.2611	0.0603	20.929	49	< .0001
stbias	-0.0128 (0.0785)	0.0308 (0.1150)	-0.0436	0.0179	-2.439	49	0.0183
genbias	-0.0496 (0.1129)	-0.0258 (0.1073)	-0.0238	0.0201	-1.185	49	0.2417
θ	0.4078 (0.232)	0.500 (0.249)	-0.0924	0.0420	-2.201	49	0.0324

Supplementary Table 3: General linear model showing individual differences in social preferences predicting early gaze-biases.

**Model predicting average early gaze-biases**						
**Predictors:**	**Statistics**					
	**b**	**SE**	**t**	**df**	**two-tailed p**	**semi-partial R^2**
Intercept	0.3734	0.0934	3.998	47	0.0002***	-
Average w_{self}	-11.3311	6.5968	-1.718	47	0.0924	0.053
Average w_{other}	-12.8192	5.2944	-2.421	47	0.0194*	0.101
Time pressure (Effect-coded: High = 1, Low = -1)	0.0733	0.0277	2.649	47	0.0109*	0.012
Average w_{self} × Time pressure	2.6368	1.9535	1.350	47	0.1836	0.003
Average w_{other} × Time pressure	-3.6561	1.5678	2.332	47	0.0240*	0.009

Supplementary Table 4: General linear model showing individual differences in social preferences interacting with attention to produce changes in generosity under time pressure.

**Model predicting changes in generosity**						
**Predictors:**		**Statistics**				
**Full model**	**Selected model**	**b**	**SE**	t_{38}	**two-tailed p**	**semi-partial R^2**
Intercept	Intercept	-0.0098	0.0280	0.351	0.727	-
Average w_{self}	Average w_{self}	-2.1066	1.4837	-1.420	0.164	0.050
Average w_{other}	Average w_{other}	-6.0767	2.8814	-2.109	0.042*	0.105
Average early gaze (+; self-biased, - : other-biased)	Average early gaze	-0.3488	0.0483	-0.722	0.475	0.014
Change in early gaze	Change in early gaze	-0.7342	0.6191	-1.186	0.243	0.036
Average w_{other} × Average early gaze	Average w_{other} × Average early gaze	-5.3781	-3.665	-1.467	0.151	0.054
Average w_{other} × Change in early gaze	Average w_{other} × Change in early gaze	1.5417	5.4673	0.282	0.780	0.002
Average early gaze × Change in early gaze	Average early gaze × Change in early gaze	0.0010	0.1636	0.006	0.995	0.000
Average w_{other} × Average early gaze × Change in early gaze	Average w_{other} × Average early gaze × Change in early gaze	33.2281	14.7726	2.249	0.030*	0.117
Average late gaze	Average late gaze	-0.0307	0.0679	0.451	0.654	0.005
Average w_{other} × Average late gaze	Average w_{other} × Average late gaze	-8.3620	6.0640	-1.379	0.176	0.048
Change in w_{other}	Change in w_{other}	3.6129	1.9966	1.809	0.078	0.079
Average w_{self} × Average early gaze						
Average w_{self} × Change in early gaze						
Average w_{self} × Average early gaze × Change in early gaze						
Average w_{self} × Average late gaze						
Average $w_{fairmess}$						
Average genbias						
Change in w_{self}						
Change in $w_{fairness}$						

Supplementary References

1. Chen, F. & Krajbich, I. Biased sequential sampling underlies the effects of time pressure and delay in social decision making. *Nat. Commun.* **9**, 3557 (2018).
2. Wiecki, T. V, Sofer, I. & Frank, M. J. HDDM: hierarchical bayesian estimation of the drift-diffusion model in python. *Front. Neuroinform.* **7**, 14 (2013).
3. Rand, D. G., Greene, J. D. & Nowak, M. A. Spontaneous giving and calculated greed. *Nature* **489**, 427–430 (2012).
4. Bouwmeester, S. *et al.* Registered replication report: Rand, greene, and nowak (2012). *Perspect. Psychol. Sci.* **12**, 527–542 (2017).
