# Supplementary material for: Attentional priorities drive effects of time pressure on altruistic choice
Source: Nat Commun. 2020 Jul 15;11:3534. doi: 10.1038/s41467-020-17326-x (PMC7363879; doi:10.1038/s41467-020-17326-x)
Supplement: Supplementary file 3 — Reporting Summary [file 41467_2020_17326_MOESM3_ESM.pdf]

nature research

Corresponding author(s): Yi Yang Teoh

Last updated by author(s): May 27, 2020

Reporting Summary

Nature Research wishes to improve the reproducibility of the work that we publish. This form provides structure for consistency and transparency in reporting. For further information on Nature Research policies, see Authors & Referees and the Editorial Policy Checklist.

Statistics

For all statistical analyses, confirm that the following items are present in the figure legend, table legend, main text, or Methods section.

n/a	Confirmed	
☐	☒	The exact sample size (n) for each experimental group/condition, given as a discrete number and unit of measurement
☐	☒	A statement on whether measurements were taken from distinct samples or whether the same sample was measured repeatedly
☐	☒	The statistical test(s) used AND whether they are one- or two-sided<br>*Only common tests should be described solely by name; describe more complex techniques in the Methods section.*
☐	☒	A description of all covariates tested
☐	☒	A description of any assumptions or corrections, such as tests of normality and adjustment for multiple comparisons
☐	☒	A full description of the statistical parameters including central tendency (e.g. means) or other basic estimates (e.g. regression coefficient) AND variation (e.g. standard deviation) or associated estimates of uncertainty (e.g. confidence intervals)
☐	☒	For null hypothesis testing, the test statistic (e.g. F, t, r) with confidence intervals, effect sizes, degrees of freedom and P value noted<br>*Give P values as exact values whenever suitable.*
☐	☒	For Bayesian analysis, information on the choice of priors and Markov chain Monte Carlo settings
☐	☒	For hierarchical and complex designs, identification of the appropriate level for tests and full reporting of outcomes
☐	☒	Estimates of effect sizes (e.g. Cohen's d, Pearson's r), indicating how they were calculated

Our web collection on statistics for biologists contains articles on many of the points above.

Software and code

Policy information about availability of computer code

Data collection	In Study 1, Replication Study 1 & 2, the stimuli were presented and responses collected using MATLAB (v2015b) and the Psychophysics Toolbox (v3.0.11) running on Windows PCs. In Study 2, we conducted the study online using Inquisit Web 5.0.14.0.
Data analysis	All statistical analyses, except for continuous eye-tracking analyses and computational model fitting, were conducted in R 3.6.0. General linear mixed effects modelling was conducted using the lme4 package (v1.1-21) with degrees of freedom estimated using the Satterthwaite method. We conducted stepwise regression as implemented using the stepAIC function from the MASS package (v7.3-51.4). Effect size statistics of R2 and partial R2 were computed using the r2beta function from the r2glmm package (v0.1.2) using standardized general variances. Continuous eye-tracking analyses were conducted using MATLAB (v2017b) and custom computational model fitting was conducted in Python (v3.6) based on kernel density estimation with SciPy (v1.4.1). Computations were parallelized using the native MATLAB Distributed Computing Server and the concurrent futures module in the Python Standard Library in Python. Supplementary model-fitting was conducted using HDDM (v0.6.0) in Python (v3.5).

For manuscripts utilizing custom algorithms or software that are central to the research but not yet described in published literature, software must be made available to editors/reviewers. We strongly encourage code deposition in a community repository (e.g. GitHub). See the Nature Research guidelines for submitting code & software for further information.

Data

Policy information about availability of data

All manuscripts must include a data availability statement. This statement should provide the following information, where applicable:

- Accession codes, unique identifiers, or web links for publicly available datasets
- A list of figures that have associated raw data
- A description of any restrictions on data availability

The experimental data that support the findings of this study is available in OSF with the identifier: https://osf.io/vf6a5/. All source data for the figures are available at the same link

Field-specific reporting

Please select the one below that is the best fit for your research. If you are not sure, read the appropriate sections before making your selection.

☐ Life sciences ☒ Behavioural & social sciences ☐ Ecological, evolutionary & environmental sciences

For a reference copy of the document with all sections, see nature.com/documents/nr-reporting-summary-flat.pdf

Behavioural & social sciences study design

All studies must disclose on these points even when the disclosure is negative.

Study description	In a quantitative study, we experimentally manipulated time pressure in a dictator game paradigm in order to measure how time pressure changes in altruistic behavior: choice and eye movements in a within-subject repeated measures design. In a second study we additionally manipulated attention to choice attributes while participants made similar altruistic choices.
Research sample	60 students (18 M/42F; Age range: 18-39) from the University of Toronto were recruited to participate in study 1 for course credit or monetary compensation for their time as a convenience sample. For study 2, we recruited 396 (206M;160F;2NB; Age range: 20-71) participants from Amazon mechanical TURK to participate in our study as a more representative sample of participants from the US and Canada. We compensated them $5 for their time. We further recruited 34 (9M/22F), 31 (15M/16F) and 49 (16M/33F) students from the University of Toronto in replication studies 1A, 1B and 2 to replicate the behavioural effects observed in the primary study (We did not collect participant age in these studies). All subjects had normal or corrected-to-normal vision and were naive to the purpose of the study. Written, informed consent was obtained from all participants and they were informed of their right to discontinue participation at any time
Sampling strategy	For study 1, replication study 1 and 2 , no sample-size calculation was performed, but the sample size was selected to match previous successful studies using a similar paradigm (Hutcherson, Bushong & Rangel, 2015). However, for study 2, we calculated sample size of 200 participants based on pilot data and sampled participants until we achieved the target after our preregistered exclusions.
Data collection	For studies 1, replication study 1 and 2, the data was collected in the lab and required participants to make keyboard responses while eye movements were recorded from their right eye using an Eyelink 1000 plus Desktop Mount (SR Research, Canada). The research assistants monitored participants from an adjacent room but were not blinded to the hypotheses of the studies. Data collection for study 2 was conducted online using Inquisit Web 5.0.14.0.
Timing	All data collection for study 1, replication studies 1 and 2 occurred between Feb 2016 to Mar 2018. We collected data for study 2 in Jan - Feb 2020.
Data exclusions	3 participants from study 1 were excluded from all analyses that involved eye-tracking due to technical difficulties with calibration of eye-movements at the time of data-collection. We additionally excluded 196 participants from study 2 based on preregistered criteria which can be found at https://osf.io/chwm3.
Non-participation	No participants declined participation
Randomization	For study 1,replication study 1 and 2, response keys (accept vs reject) were counterbalanced across participants by subject ID such that participants with ID#s that were odd were assigned to a condition where acceptance was the option on the left and rejection on the right, while participants with ID#s that were even were assigned to a condition where acceptance was the option on the right and rejection on the left.<br><br>For the study 1 and replication study 2, stimuli presentation of value information pertaining to self and other to the left and right side of the screen was counterbalanced between participants by subject ID such that ID#s 1,2,5,6,9,10,etc. viewed self-information on the left and other-information on the right while IDs 3,4,7,8,11,12,...etc viewed self-information on the right and other-information on the left. In replication studies 1A and 1B, the information presented were counterbalanced similarly in terms of the vertical order it was presented in (top, bottom).<br><br>For study 2, randomization of the experiment can be found on the linked OSF page in the manuscript.

Reporting for specific materials, systems and methods

We require information from authors about some types of materials, experimental systems and methods used in many studies. Here, indicate whether each material, system or method listed is relevant to your study. If you are not sure if a list item applies to your research, read the appropriate section before selecting a response.

Materials & experimental systems

n/a	Involved in the study
☒	☐ Antibodies
☒	☐ Eukaryotic cell lines
☒	☐ Palaeontology
☒	☐ Animals and other organisms
☐	☒ Human research participants
☒	☐ Clinical data

Methods

n/a	Involved in the study
☒	☐ ChIP-seq
☒	☐ Flow cytometry
☒	☐ MRI-based neuroimaging

Human research participants

Policy information about studies involving human research participants

Population characteristics	See above.
Recruitment	Students were recruited from the participant pool at the University of Toronto for Study 1. Participants in this study may not be representative of the general population due to their similarity in socio-economic demographics as university students. Participants in Study 2 were recruited from Amazon mechanical TURK which may be more representative of the general population than in Study 1 but limited to individuals who have ready access to internet and technology. These differences should not heavily impact our results given the fundamental psychological processes being investigated here and the robust replication of finding across these different populations.
Ethics oversight	The study was approved by the Research Ethics Board at the University of Toronto.

Note that full information on the approval of the study protocol must also be provided in the manuscript.
